# An Experimental and Numerical Investigation to Characterize an Aerospace Composite Material with Open-Hole Using Non-Destructive Techniques

**DOI:** 10.3390/s20154148

**Published:** 2020-07-26

**Authors:** Norberto Feito, José Vicente Calvo, Ricardo Belda, Eugenio Giner

**Affiliations:** 1 Centre of Research in Mechanical Engineering—CIIM, Department of Mechanical Engineering and Materials, Universitat Politècnica de València, Camino de Vera s/n, 46022 Valencia, Spain; ribelgon@upv.es (R.B.); eginerm@mcm.upv.es (E.G.); 2 Department of Mechanical Engineering, Universidad Carlos III de Madrid, Avda, Universidad 30, 28911 Leganés, Madrid, Spain; jocalvoo@ing.uc3m.es; 3 Networking Biomedical Research Centre in Bioengineering, Biomaterials and Nanomedicine (CIBER-BBN), Universitat Politècnica de València, Camino de Vera s/n, 46022 Valencia, Spain

**Keywords:** CFRP, fatigue, digital image correlation, infrared thermography, open hole

## Abstract

In this study, the open-hole quasi-static tensile and fatigue loading behavior of a multidirectional CFRP thick laminate, representative of laminates used in the aerospace industry, is studied. Non-destructive techniques such as infrared thermographic (IRT) and digital image correlation (DIC) are used to analyze the behavior of this material. We aim at characterizing the influence of the manufacturing defects and the stress concentrator through the temperature variation and strain distribution during fatigue and quasi-static tests. On the one hand, the fatigue specimens were tested in two main perpendicular directions of the laminate. The results revealed that manufacturing defects such as fiber waviness can have a major impact than open-hole stress concentrator on raising the material temperature and causing fracture. In addition, the number of plies with fibers oriented in the load direction can drastically reduce the temperature increment in the laminate. On the other hand, the quasi-static tensile tests showed that the strain distribution around the hole is able to predict the crack initiation and progression in the external plies. Finally, the experimental quasi-static tests were numerically simulated using the finite element method showing good agreement between the numerical and experimental results.

## 1. Introduction

Carbon Fiber Reinforced Polymers (CFRP) composites have a wide range of applications in the automobile and aerospace industries due to their superior mechanical properties combined with reduced density and good resistance to corrosion and fatigue. They are commonly used at critical areas of engineering structures due to their high specific strength and stiffness [1]. Since composite laminates are often used in load-carrying structures, an important issue is to evaluate the allowable design values of composite structures which considers various defects and damages [2,3].

Fiber reinforced polymer materials can be manufactured by several processes such as liquid molding, compression molding, resin infusion and injection molding. All these processes belong to autoclave manufacturing methods, and each of them produces different manufacturing fiber, waviness defects being one of the most relevant [4,5,6]. The waviness is produced due to the axial compression of the fibers by the non-uniform pressure distribution between films and has a predominant effect on mechanical properties such as tensile, compressive, flexural and fatigue strength, being one of the most studied defects in the literature [7,8,9,10,11].

The use of these materials in the manufacturing of large structures, such as aircrafts, requires mechanical joining of the parts using rivets or bolts. To generate the holes for these joints, the most widely used machining process is drilling [12,13,14]. However, due to the low machinability of the CFRP materials caused by the abrasive character of the fiber reinforcement, the workpiece is susceptible to experience drilling induced damage, delamination being the prevalent damage [15,16,17]. The life in service of the component can be affected by this problem [18,19,20,21] and is still a challenge for the aerospace industry to reduce the induced damage during machining [14,22,23].

To characterize the behavior of the composite, the open-hole tension (OHT) and open-hole compression (OHC) tests are often used. During the last decades, relevant works focused on understanding the damage mechanism of laminates subjected to longitudinal loads. In the quasi-static OHT studies, different sizes and lay-ups were analyzed. Three distinctive failure modes are commonly found in the specimens: brittle failure, pull-out, and delamination. Delamination is the main failure affecting the in-plane strength, failure mechanism and hole size effect [24,25,26,27,28]. For the OHC tests, failure was initiated as matrix cracking, but increasing the load, fiber kinking and delamination take dominant roles, especially at the edges of the hole. When damage reached a critical condition, the laminate fails catastrophically [29,30,31,32,33]. The open hole geometry is also used in fatigue studies [34,35,36,37,38], where it is proved that longitudinal strain, stiffness and surface temperature can provide valuable information regarding damage progression, fatigue life, and also for predicting notched fatigue performance.

Non-destructive methods are an effective tool to detect and control the damage evolution before failure caused by manufacturing defects or fatigue loading. To monitor the damage state in polymer matrix laminated structures, many characterization techniques are available nowadays, but the most widely used techniques are ultrasonic C-scan [36,39], X-ray radiography [33,40,41,42], acoustic emission [43,44], ultrasonic testing [45,46], infrared thermography (IRT) [47,48,49] and Digital Image Correlation (DIC) [26,37,50,51]. The non-destructive techniques also help us to develop and validate damage progression models used to predict the damage occurring prior to failure [27,28,52]. In this work, we use only IRT and DIC techniques.

During cycle loading of composite structures, material temperature varies as a function of material kinematics. The heating of the CFRP composite must be controlled because heat can affect the mechanical behavior and instigate chemical changes to the polymer matrix. Infrared thermography allows for measuring the surface temperature variation of the emitting body. This method can be applied in a passive or active mode: the first is generally applied on materials, which experience a different temperature than the surrounding materials; the second needs an external stimulus to induce a surface temperature variation, which can be heat or a mechanical source [53]. The evaluation and growth of the damage is possible in active mode under fatigue testing [46,47,54] as the evaluation of fatigue strength/durability/limit [55,56,57]. Despite the versatility of this methodology to be applied over different types and geometries [37,52,58,59,60], not many studies have focused on the open hole thick multi-directional laminates.

The Digital Image Correlation (DIC) is one of the optical methods applied to obtain accurate displacement and strain distributions on the surface of materials. By this method, 2D and 3D displacement fields could be obtained, but two or more cameras are required in the 3D case [61]. DIC is applied to different problems in composites, such as 3D woven carbon/epoxy composite to characterize the strain field distribution during quasi-static tests [37] or to locate the fracture initiation in the GFRP tensile tests [62]. It has been also applied to fatigue studies, for example, to determine the interlaminar shear stress distributions and their variations with the number of cycles for carbon/epoxy composites [51] or to measure deformations on the pin surface for low cycle fatigue loads [63].

Despite the extensive literature, damage mechanisms and microscopic damage characteristics of open-hole thick laminates are still not fully described. In this study, the behavior of a thick CFRP laminate used in the aircraft industry is investigated. The purpose of this paper is to discuss damage detection and progression using non-destructive techniques during quasi-static and fatigue tests in specimens with stress concentrators. Among the existing techniques, two are investigated in this study: Infrared Thermography and Digital Image Correlation. Post-fatigue damaged samples were also analyzed using an optical microscope. Finally, the experimental tests are numerically simulated using the finite element method (FEM) in conjunction with a progressive damage analysis.

## 2. Materials and Experimental Setup

The material studied in this work is currently used in industry and is part of multiple structural elements of commercial aircraft. Experimental tests were performed on CFRP specimens cut from quasi-isotropic laminate plates made of 54 unidirectional carbon/epoxy plies stacked with different orientations (0°, 45°, −45°, 90°). The total thickness of the laminate is 9.7 mm and the resin content is 34%.

**Figure 1:**
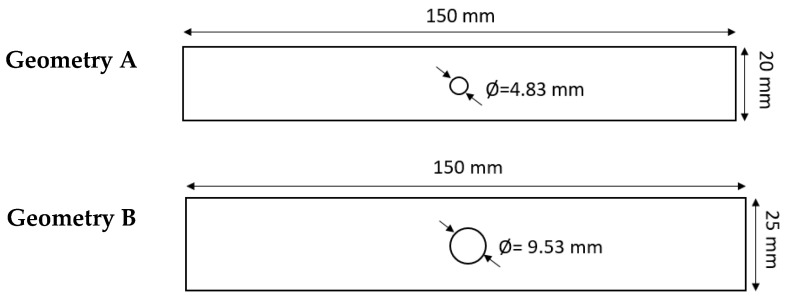
Geometries of the CFRP specimens. Samples of each geometry were cut considering both the longitudinal and transverse directions of the original laminate plate.

Two different geometries are used in the study, depicted in Figure 1. Samples of these geometries were prepared for both the longitudinal and transverse direction of the original laminate plate material. The hole and the external perimeter were cut using an abrasive waterjet cutting technique.

Two non-destructive techniques were applied to analyze the mechanical behavior of these specimens under different load conditions: infrared thermography method for the fatigue study and digital image correlation method for the tensile test study. A hydraulic testing machine, INSTRON 8801, with a load cell of 100 kN, was used for both the static and fatigue tests. The next sections explain the set-up for each case.

### 2.1. Infrared-Monitored Fatigue Tests

Infrared thermography (IRT) is a non-destructive technique (NDT) widely used in the mechanical and structural fields for contactless measurement of the surface temperature distribution of an object. This technique reveals internal defects and material inhomogeneity of components, especially in the aeronautical field, because it allows quick inspections of large areas.

Based on thermoelastic theory, the temperature of a material changes when the material changes volume due to mechanical work. The temperature variation can be related to the stress change in an adiabatic environment using Equation (1), where Δ*T* is the surface temperature variations of the sample during a fatigue cycle; T is room temperature; ρ is the density; $C_{p}$ is the specific heat capacity at constant pressure; α_11_ and α_22_ are the surface coefficients of thermal expansion in 1 and 2 directions; Δσ_1_ and Δσ_2_ are the amplitudes of the principal stresses at the surface [64]. All these parameters can be found in the literature [65,66].

(1) $$\Delta T=-\frac{T}{C_{p}\rho }\left(\alpha_{11}\Delta \sigma_{1}+\alpha_{22}\Delta \sigma_{2}\right)$$

Usually, the loading process can be treated as adiabatic if the fatigue loading frequency is higher than 5 Hz. Therefore, the temperature variation during a cyclic load is related to the stress change. The development of the stresses during the fatigue loading can be considered as an indicator of damage growth [67].

**Figure 2:**
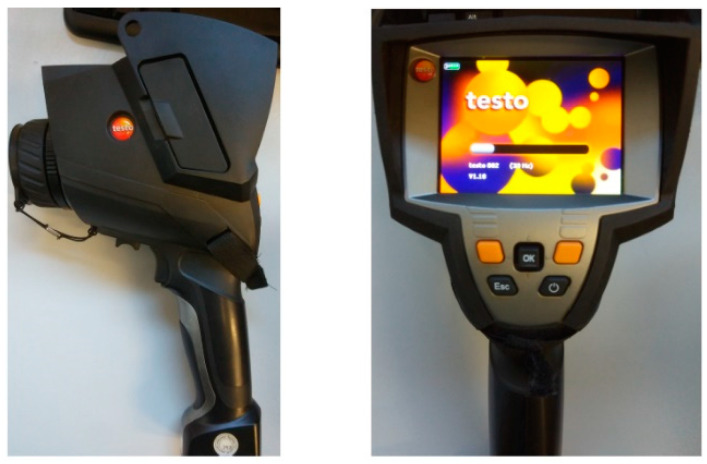
IR camera Testo 882 to measure surface temperature variation used during fatigue tests.

For this analysis, all tests were conducted at room temperature. During fatigue testing, infrared images of the samples surfaces were acquired using an IR camera (Testo 882, Titisee-Neustadt, Germany) to measure the variation of the specimen surface temperature. This camera is shown in Figure 2. The IR camera pixel resolution of 320 × 240 and the temperature sensitivity of 50 mK was sufficient to monitor the temperature variation accurately.

**Figure 3:**
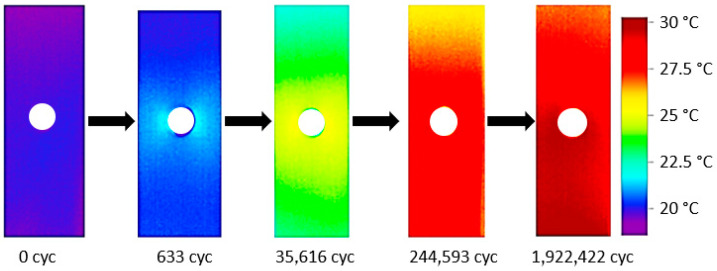
Example of the surface temperature evolution during fatigue test.

This part of the study was carried out using specimens oriented in longitudinal and transversal orientations of the original laminate plate material. The fatigue tests were performed under load control mode with a σ_max_ = 60 kN, a σ_min_ = 0 kN and a frequency of 10 Hz to minimize non-adiabatic effect and reduce testing time [64]. The limit of cycles was established at 2 million. Figure 3 shows an example of the temperature variation measurement in a longitudinal specimen during fatigue testing with the geometry A.

### 2.2. Digital Image Correlation Monitored Quasi-Static Tests

**Figure 4:**
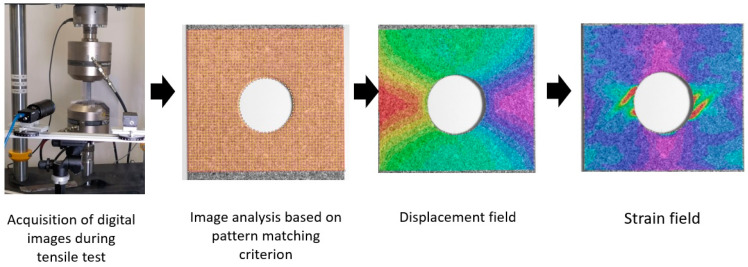
Scheme of digital image correlation (DIC) procedure.

The Digital Image Correlation technique (DIC) was used to obtain the strain field on the up layer of the laminate during quasi-static tests. This non-destructive methodology is an optical displacement measure technique that employs image pattern tracking for accurate 2D or 3D surface deformation measurements during testing. In this work, 2D tracking was used. The DIC technique divides the region of interest (ROI) in squared faces to track their displacement based on an image pattern matching criterion and compares each deformed image with either the reference or the previous one. A ROI that covered the whole area around the hole of the specimen was defined. A scheme of the DIC technique application process is shown in Figure 4. As can be seen, the DIC technique enables the detection of strain concentration zones in the analyzed structures, and it is also possible to identify the regions where cracking is initiated.

**Figure 5:**
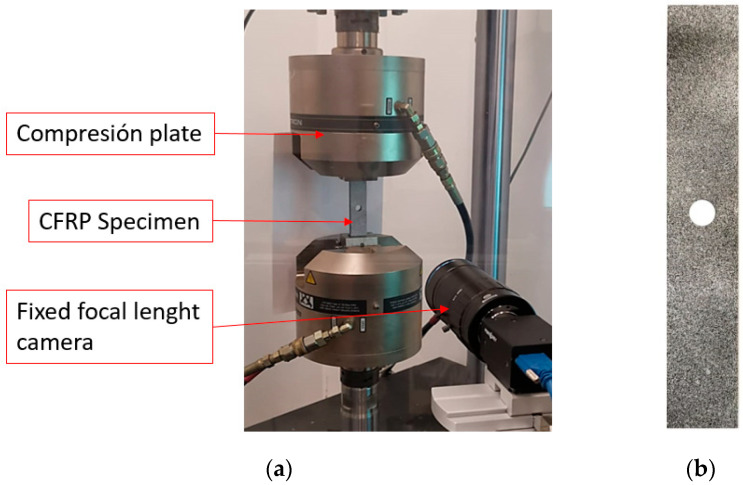
(**a**) Experimental testing and imaging set-up; (**b**) tensile specimen with speckled pattern.

Images were taken with a high resolution fixed focal lens (HF7518V-2, Myutron, Tokyo, Japan) and extension rings of 10 mm (focal length of 65 mm). In order to apply DIC, the upper layer surface of the specimen was speckled using randomly distributed black and white spray paints to increase contrast, as shown in Figure 5b. Moreover, the quality of the pattern was inspected using VIC-2D Digital Image Correlation software (v.6.0.2 Correlated Solutions Inc., Irmo, SC, USA) to verify optimum speckle pattern on the surface of the specimens. A perpendicular relative position between camera and specimen was ensured to acquire images and to avoid out-of-plane displacements during testing, as shown in Figure 5a. All tests were performed under displacement control mode with quasi-static conditions, and an applied displacement rate of 2 mm/min following the D5766/D5766M standard [68]. The force-displacement response data were registered. No pre-load was applied, and all tests were continued until the global failure of the specimens.

A facet size (the grid which divides the ROI) of 23 pixels and a step size (the spacing between control points) of 5 pixels were defined for tracking the speckle pattern during the mechanical test. A zero-normalized squared difference (ZNSSD) pattern-matching criterion was used to perform displacement correlation. Reference measurements were taken in a single image mode to enable DIC calibration. The results obtained were calibration deviation = 0.008 pixels (limit value: 0.011 pixels) and scale deviation = 0.16 µm (limit value: 0.22 µm).

The images acquired at the test were analyzed with the abovementioned software to estimate the surface displacement and engineering strain fields. Macro damages such as cracking were also examined from the obtained strain maps.

## 3. Numerical Model Implementation

**Figure 6:**
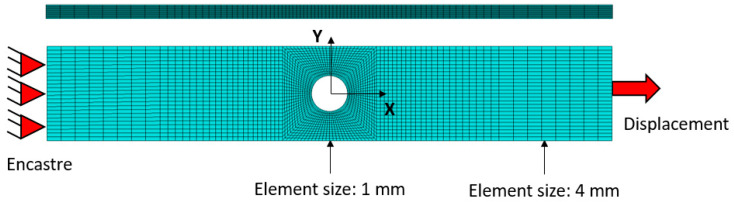
Representation of the finite element model generated considering the test conditions. A high level of discretization was defined at the stress concentration region.

Finite element models of each configuration were developed using ABAQUS/Standard code, aimed at simulating the quasi-static tests described previously. Each model reproduces the multidirectional carbon fiber component described earlier. The laminate is implemented with 3D solid elements C3D8R (8 node-linear brick, reduced integration and hourglass control), with a size of 1 mm close to the hole zone and 4 mm far from this area. One element per ply is defined through the thickness. The symmetry plane in the stacking sequence is considered, hence only half of the laminate is defined (27 plies) to save computational time. The displacement of the workpiece is restricted at one side and a fixed velocity of 2 mm/min is applied to the opposite side simulating the same experimental conditions. All the layers are tied. The scheme of the full model, including boundary conditions, is presented in Figure 6.

### Material Behavior

**Figure 7:**
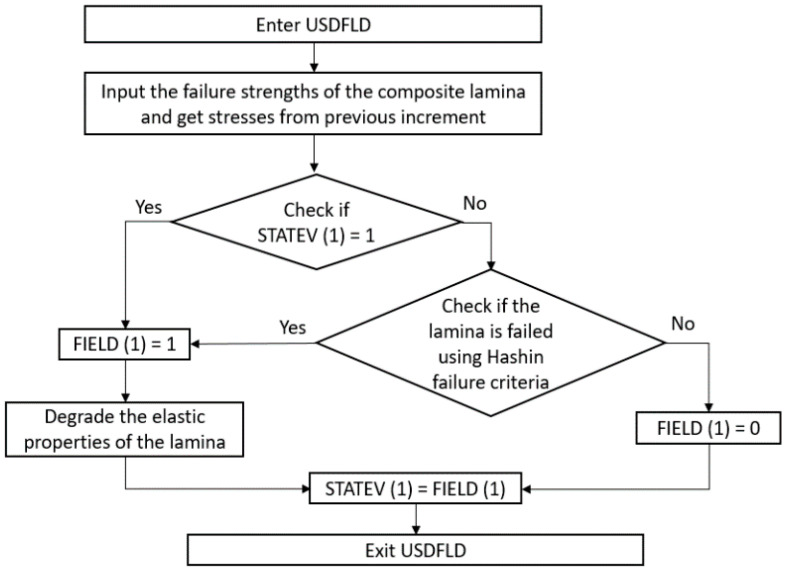
Flowchart of the Progressive Damage Model implemented in the Abaqus user subroutine USDFLD.

The plies have been modeled assuming an elastic behavior until failure due to their high strength. The failure of the plies has been defined using the Hashin criterion [69], which considers matrix cracking (traction failure mode Equation (2) and compression failure mode Equation (3)), fiber breakage in tension—Equation (4)—and fiber micro-buckling in compression—Equation (5). In these equations, capitalized magnitudes refer to the corresponding failure strengths. The damage behavior has been implemented using an Abaqus user subroutine USDFLD, which follows the flowchart in Figure 7.

(2) $${\left(\frac{\sigma_{22}+\sigma_{33}}{Y_{t}}\right)}^{2}+\frac{\tau_{23}^{2}-\sigma_{22}\sigma_{33}}{S_{t}^{2}}+\frac{\tau_{12}^{2}+\tau_{13}^{2}}{S_{l}^{2}}=1$$

(3) $$\left[{\left(\frac{Y_{c}}{2\;S_{t}}\right)}^{2}-1\right]\frac{\sigma_{22}+\sigma_{33}}{Y_{c}}+{\left(\frac{\sigma_{22}+\sigma_{33}}{2\;S_{t}}\right)}^{2}+\frac{\tau_{23}^{2}-\sigma_{22}\sigma_{33}}{S_{t}^{2}}+\frac{\tau_{12}^{2}+\tau_{13}^{2}}{S_{l}^{2}}=1$$

(4) $${\left(\frac{\sigma_{11}}{X_{t}}\right)}^{2}+\frac{\tau_{12}^{2}+\tau_{13}^{2}}{S_{l}^{2}}=1$$

(5) $$\frac{-\sigma_{11}}{X_{c}}=1$$

## 4. Results and Discussion

### 4.1. Thermal Analysis Using Infrared Thermography

**Figure 8:**
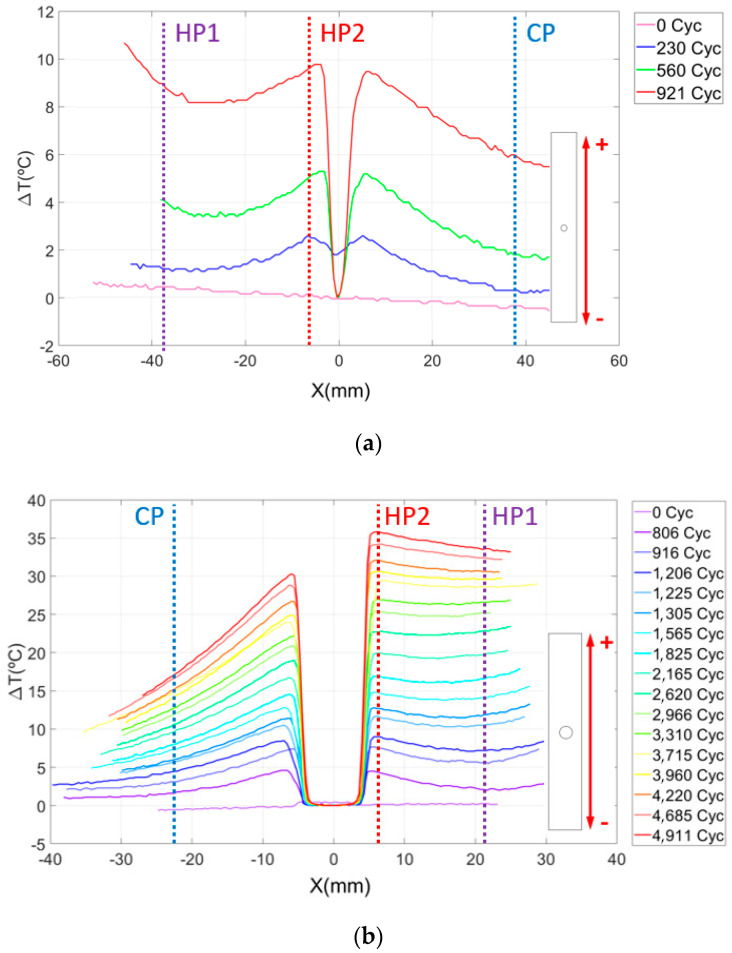
Evolution of the surface temperature variation as a function of the number of load cycles in specimens cut at longitudinal direction for (**a**) geometry A and (**b**) geometry B.

The results of the thermographic analysis in CFRP specimens with-open hole are now presented. Figure 8 presents a plot of the evolution of the temperature increment in the longitudinal middle section of the specimen cut in the direction of the fibers at 0° as a function of the number of load cycles. The variation in the temperature in the *Y* axis is calculated as *T*_(*x*)_—*T*_0_ where *T*_(*x*)_ is the temperature of the material at *x* distance from the center of the hole in a vertical direction and *T*_0_ is the initial temperature of the specimen. This expression defines the temperature origin at the hole and is used as an indicator of damage progression.

It is relevant to highlight two phenomena observed in the graphics. The first is the increasing temperature as we approach the hole from the sides of the material. This effect is observed for any cycle and is a consequence of the stress concentrator, which redistributes the strain and stress around the hole [70,71].

**Figure 9:**
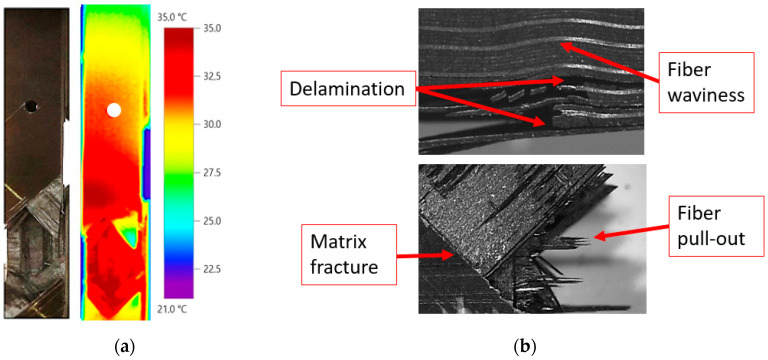
(**a**) Specimen temperature at breakage time for geometry A and (**b**) failure mechanisms observed under optical microscope after breakage.

The second phenomenon is related to the fact that the variation of the temperature at one side of the specimen increases faster with the number of cycles than at the other side. This can be clearly observed in Figure 8 and Figure 9. This thermoelastic response breaks the temperature symmetry along the material characteristic for a low number of cycles, resulting in a non-symmetric thermoelastic response unexpected in simple uniaxial tension. We hypothesize that it is probably caused by the fiber waviness of the laminate. Fractured specimens observed under optical microscope revealed that the breakage of the material is affected by these manufacturing defects. Thus, fracture is located near the area with important ply misalignment, as observed in Figure 9b.

Several authors in the literature have studied the influence of this type of defect [72,73]. The ply waviness has proved to be a significant parameter which produces failure at a much lower number of cycles than what would be expected by specimen without ply waviness. Moreover, these studies reveal that fiber waviness acts as local shear stress raiser throughout the thickness, so the effect of fatigue contributes to the delamination of the material much faster, generating high temperature rise at those regions inside the material [74].

**Figure 10:**
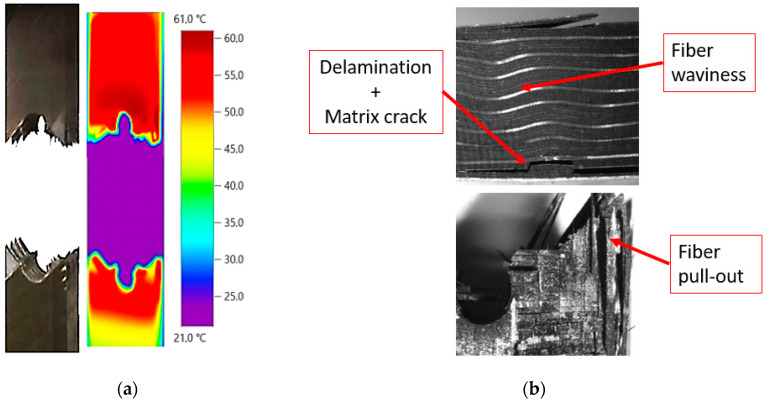
(**a**) Specimen temperature at breakage time for geometry B and (**b**) failure mechanisms observed under optical microscope after breakage.

After analysis of the results, it is observed that waviness produces an area of higher temperature where failure occurs. The combination of the local undulation with the stress raiser due to the hole leads to a more critical condition than at the opposite side. For the case shown in Figure 9a, the load was around 85% of the static strength, causing the breakage close to the area with manufacturing defect, as mentioned. For the geometry B, the load was around 70% of the static strength, causing a delamination less severe in the fiber waviness area as shown in Figure 10b. In this case, the fracture was located through the hole (see Figure 10a). The difference between the two specimens was also observed in Figure 8. In these plots, the maximum temperature is reached in the area where final catastrophic failure was located.

To quantify the influence that both stress raisers (open hole and fiber waviness) have on the material temperature distribution under fatigue conditions, Table 1 and Table 2 show the absolute temperature of the surface material at points close to the stress concentrators, named Hot Points, and the temperature in an area far from both critical points, named the Cold Point. These points have been marked with a line in Figure 8 as HP1 and HP2 and CP, respectively.

On the one hand, the fiber waviness (HP1) raises the laminate surface temperature by 41% and 95% before breaking for geometries A and B, respectively. On the other hand, the surface temperature around the hole (HP2) increases by 35% and 111% for the same geometries. This means that the damage pattern of the material is also characterized by a greater temperature gradient at these regions, as shown in Figure 9 and Figure 10. In the cold area, the surface temperature value increases only by 27% and 32% for geometries A and B, which points out the clear influence of the waviness and hole on temperature variation, due to the high stresses detected. In this area, minor damage was observed at the microscope.

**Figure 11:**
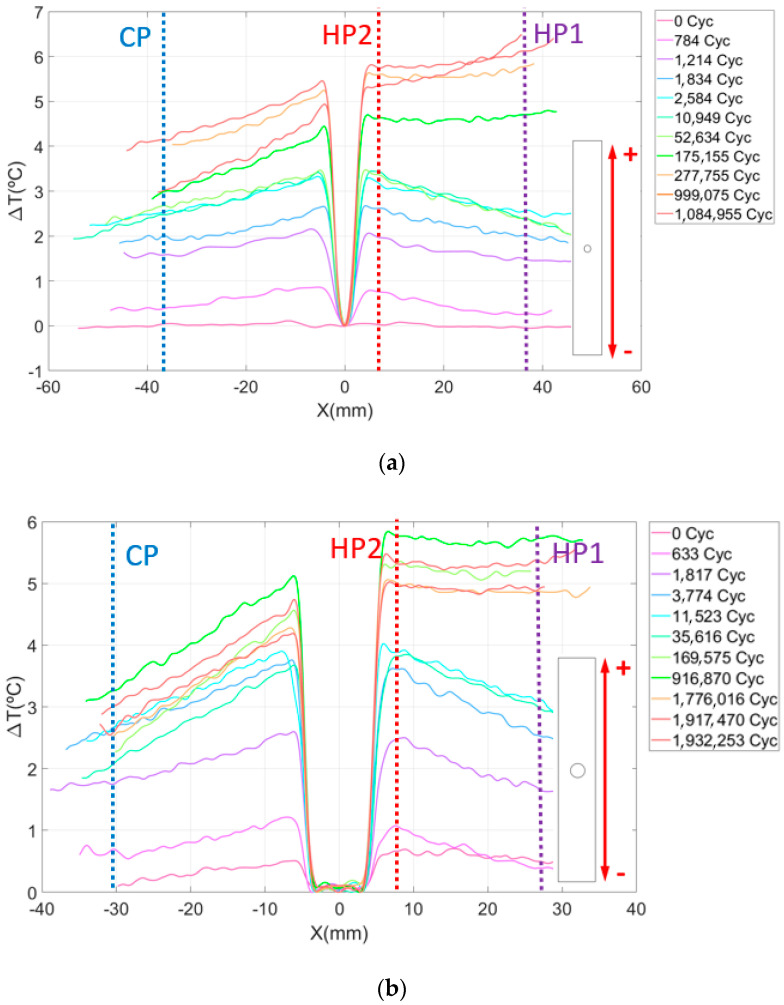
Evolution of the surface temperature variation as function of the number of load cycles in specimens cut at transversal direction for (**a**) geometry A and (**b**) geometry B.

For the specimens cut in perpendicular direction to the plies at 0°, the damage initiation and progression scheme was similar to the previous samples. The results for these configurations are presented in Figure 11. In this case, none of the tests reached breakage because the number of plies oriented in the load direction is greater than the number of plies oriented at 0°, which increases the laminate resistance. For the same reason, the surface temperature increment is lower than the one detected in the previous specimens cut in longitudinal direction to the plate. In both cases, the load amplitude was kept, and the cycle limit was fixed at 2,000,000. Nonetheless, it is easy to identify the areas where the specimen presents defects because the surface temperature starts to increase faster than in other laminate areas. In addition, we can see that, by increasing the number of plies oriented in the traction direction, the presence of damage caused by waviness is delayed with the number of cycles.

Table 3 and Table 4 show the surface temperature of the specimens measured for the Hot and Cold control points. In this case, increments of 35.7% and 40.9% were observed for the fiber waviness defect and 26% and 23% for the open hole. For both cases, the temperature around the open hole is slightly higher at the beginning of the test. However, when the number of cycles increases, the temperature in the HP2 exceeds the temperature of the HP1 until the end of the test. This is probably an indicator of the failure initiation due to a fiber waviness [74]. Measured temperature at the cold point increases only by 19.8% and 23% for geometries A and B, respectively.

### 4.2. Damage Analysis Using DIC Technique

**Figure 12:**
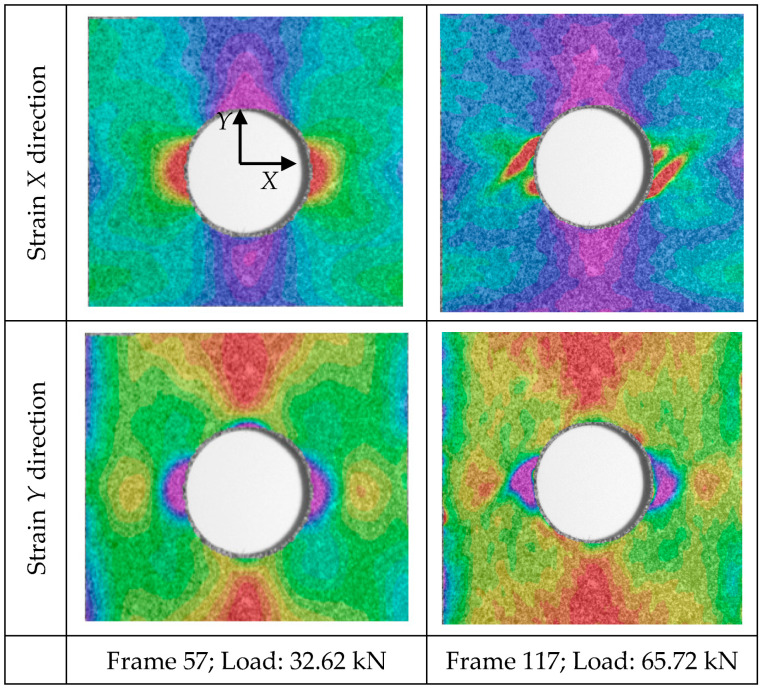
Strain distribution in *X* and *Y* directions for two different load steps.

Results from the quasi-static tensile test, analyzed through the application of Digital Image Correlation, are discussed in this section. Figure 12 presents the engineering strain distribution in *X* and *Y* directions for different load steps of the test for a specimen of geometry B. The DIC technique clearly detects the strain distribution due to the stress raiser during testing. Moreover, it predicts the highly strained areas where fracture is prone to develop; see for example the inclined highly strained distribution surrounding the hole in Figure 12 (top right).

Under small loads, the strain distribution around the open hole exhibited a pattern similar to the common pattern of an isotropic sample. As load increases, this pattern is modified because surface cracks appear (visually observed) in the open-hole region and increases the engineering strain in this area. The damage evolution observed in Figure 12 involves matrix splitting in the +45° ply, propagating from the hole edge at the location where the fiber direction is tangential to the hole. This damage pattern is also observed in similar studies with thin laminates [28,36].

**Figure 13:**
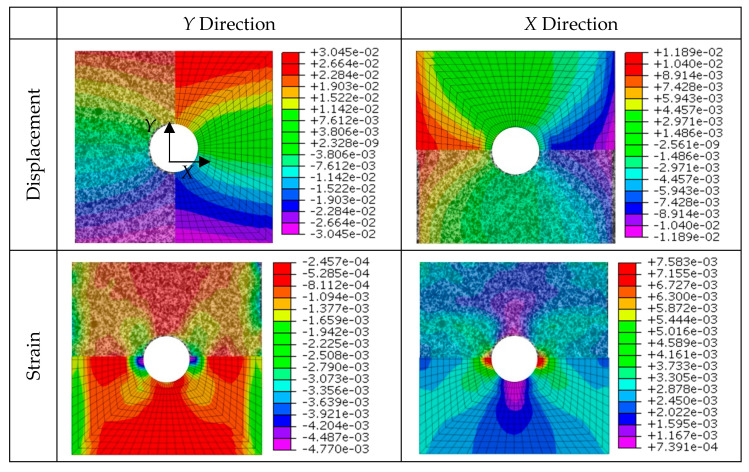
Comparison of displacement and strain fields between DIC estimation and numerical prediction for geometry A.

**Figure 14:**
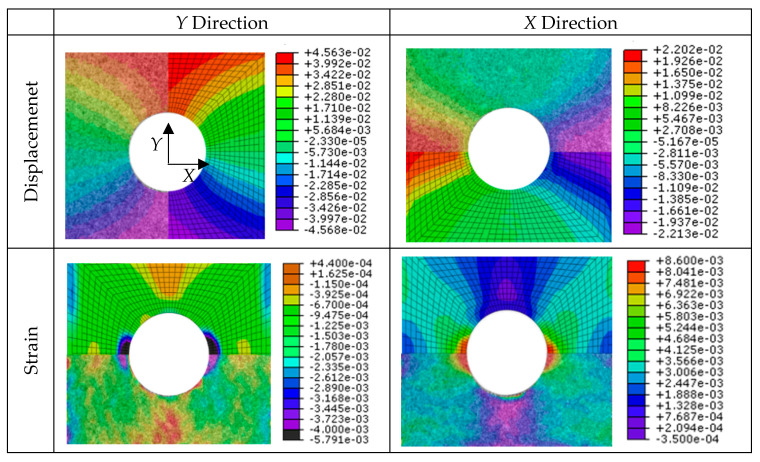
Comparison of displacement and strain field between DIC estimation and numerical prediction for geometry B.

On the other hand, two finite element models that reproduce the static tensile testing were developed. They were validated against the experimental predictions of DIC technique. Specifically, both the displacement and strain fields have been compared for geometry A (Figure 13) and geometry B (Figure 14) configurations. It can be observed that the distribution of the field is very similar for both cases and in both directions, which validates the numerical models developed and points out the effectiveness of DIC technique for surface displacement analysis.

The model predicts the displacement value with an error of 4.2%, which is acceptable. The results of the maximum and minimum displacement values obtained are presented in the following Table 5 and Table 6 for geometries A and B, respectively. Results are shown for three different instants from the start of the test. The minimum and maximum strain values have also been compared, obtaining similar results for the instants shown in Table 7 and Table 8.

**Figure 15:**
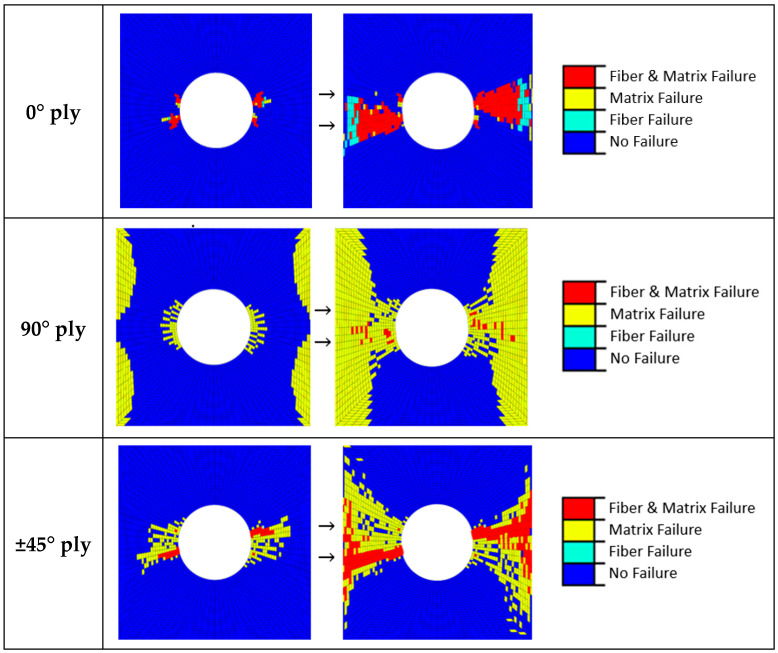
Failure patterns for different oriented plies of the CFRP laminate.

Finally, a damage study with the numerical model was carried out. Figure 15 presents the damage evolution for plies of the laminate oriented at 0°, 90° and 45°. The damage is induced with increasing the displacement in one side of the specimen. Plies at 0° are oriented in the loading direction. For this case, a combined fiber-matrix failure starts at the hole and propagates perpendicular to the loading direction towards the border of the laminate. This damage accumulation strongly reduces the stress concentration introduced by the hole, and the fiber failure occurs in the simulation (see Figure 15; top).

For the plies not oriented in the load direction, the majority of the damage comprises the matrix cracking of the 90° plies. In this case, fibers do not undergo traction conditions and the matrix around the hole and the free edges suffer isolated damage (see Figure 15; middle). There is also some matrix cracking in the ±45° directions and some fiber failure. These plies initiate the matrix cracking. As the load is further increased, the damage grows across the width of the specimen from the hole in a ‘‘zone of influence”, bounded by ±45° (see Figure 15; bottom). Finally, a catastrophic failure happens. The sequence of damage events are similar to previous static models [52].

## 5. Conclusions

This work was concerned with non-destructive techniques to characterize the influence of the fiber waviness defect and the stress concentrator in a thick CFRP material representative of those used in the aircraft industry.

In the first place, an infrared camera captured the surface temperature distribution during fatigue loading testing. The specimens were cut in the two main perpendicular directions of the laminate. Based on the temperature gradient distribution maps, it was observed that the fiber waviness generated during the manufacturing process can produce a more severe damage in the material than the damage generated by the open hole. The influence of the waviness defect became more relevant when the load applied was close to the limit load. The temperature (and consequently the damage) grew more rapidly in this area of the specimen than around the hole. Further investigation in the testing of multidirectional composites with both stress concentrators (fiber waviness and open hole) can be beneficial, including changes in the test frequency. The combination of both phenomena was also observed in specimens with more plies oriented in the load direction, whose fatigue testing exceeded 2,000,000 cycles. For those cases, surface temperature was reduced by 84–89% at the hottest points of the material, which means that the damage generated by the waviness can be reduced as a function of the number of plies aligned with the load.

In the second place, the digital image correlation technique (DIC) was used to obtain the strain field in the surface material during quasi-static tensile test. The upper surface layer of the specimen was speckled using randomly distributed black and white spray paints to increase contrast and a camera was used to acquire images during testing. Results revealed that the strain distribution around the hole is able to predict the crack initiation and progression in the external plies of the CFRP laminate, establishing a work-load limit for the life in service of the material. This information can be useful to avoid the catastrophic failure of a structure.

Finally, the experimental quasi-static tests were numerically simulated using the finite element method. Failure was implemented using Hashin criterion, which differentiates between matrix and fiber failure modes. Results showed good agreement between the numerical and DIC results. The model was also used to predict the damage sequence in the thick laminate. It was observed that, initially, damage starts to propagate from the hole to the free edge of the specimen in terms of matrix cracking and delamination bounded by ±45 plies. Finally, the catastrophic failure occurs with a high dispersion matrix crack damage in the 90° plies.

## Figures and Tables

**Table 1:** Surface temperature for control points of the specimen cut in transversal direction with geometry A.

**Number of Cycles:**	230	560	921
**Hot Point 1 (Waviness)**	29.2 °C	36.9 °C	41.2 °C
**Hot Point 2 (Hole)**	24.5 °C	29.5 °C	33.1 °C
**Cold Point**	20.0 °C	23.5 °C	25.4 °C

**Table 2:** Surface temperature for control points of the specimen cut in transversal direction with geometry B.

**Number of Cycles:**	806	1225	2165	3960	4911
**Hot Point 1 (Waviness)**	29.4 °C	40.0 °C	47.4 °C	54.2 °C	57.4 °C
**Hot Point 2 (Hole)**	28.7 °C	36.1 °C	43.8 °C	54.8 °C	60.8 °C
**Cold Point**	21.8 °C	24.5 °C	26.2 °C	27.7 °C	28.8 °C

**Table 3:** Surface temperature for control points of the specimen cut in transversal direction with geometry A.

**Number of Cycles:**	1834	52,634	184,666	277,093	1,084,913
**Hot Point 1 (Waviness)**	20.4 °C	23.0 °C	25.3 °C	27.4 °C	27.7 °C
**Hot Point 2 (Hole)**	21.5 °C	24.2 °C	25.6 °C	27.0 °C	27.1 °C
**Cold Point**	20.2 °C	21.6 °C	22.0 °C	24.0 °C	24.2 °C

**Table 4:** Surface temperature for control points of the specimen cut in transversal direction with geometry B.

**Number of Cycles:**	1817	35,616	169,575	916,870	1,932,253
**Hot Point 1 (Waviness)**	21.5 °C	23.0 °C	26.5 °C	28.4 °C	30.3 °C
**Hot Point 2 (Hole)**	23.3 °C	25.5 °C	26.3 °C	28.1 °C	29.3 °C
**Cold Point**	21.1 °C	22.0 °C	22.2 °C	24.2 °C	26.0 °C

**Table 5:** Maximum and minimum displacement values for geometry A.

		Instant 1		Instant 2		Instant 3	
	Value	DIC	FEM	DIC	FEM	DIC	FEM
***X*** **direction**	Max	4.70 × 10^−3^	4.79 × 10^−3^	9.00 × 10^−3^	9.43 × 10^−3^	1.08 × 10^−2^	1.15 × 10^−2^
	Min	−4.65 × 10^−3^	−4.79 × 10^−3^	−8.70 × 10^−3^	−9.43 × 10^−3^	−1.06 × 10^−2^	−1.15 × 10^−2^
***Y* direction**	Max	1.21 × 10^−2^	1.21 × 10^−2^	2.50 × 10^−2^	2.38 × 10^−2^	3.10 × 10^−2^	2.90 × 10^−2^
	Min	−1.22 × 10^−2^	−1.21 × 10^−2^	−2.55 × 10^−2^	−2.38 × 10^−2^	−3.10 × 10^−2^	−2.90 × 10^−2^

**Table 6:** Maximum and minimum displacement values for geometry B.

		Instant 1		Instant 2		Instant 3	
	Value	DIC	FEM	DIC	FEM	DIC	FEM
***X*** **direction**	Max	8.40 × 10^−3^	8.42 × 10^−3^	1.44 × 10^−2^	1.49 × 10^−2^	2.12 × 10^−2^	2.12 × 10^−2^
	Min	−8.30 × 10^−3^	−8.46 × 10^−3^	−1.42 × 10^−2^	−1.49 × 10^−2^	−2.08 × 10^−2^	−2.13 × 10^−2^
***Y*** **direction**	Max	1.80 × 10^−2^	1.76 × 10^−2^	3.15 × 10^−2^	3.09 × 10^−2^	4.55 × 10^−2^	4.41 × 10^−2^
	Min	−1.82 × 10^−2^	−1.76 × 10^−2^	−3.20 × 10^−2^	−3.10 × 10^−2^	−4.65 × 10^−2^	−4.42 × 10^−2^

**Table 7:** Maximum and minimum strain values for geometry A.

		Instant 1		Instant 2		Instant 3	
	Value	DIC	FEM	DIC	FEM	DIC	FEM
**Maximum Principal Strain**	Max	2.68 × 10^−3^	2.96 × 10^−3^	5.55 × 10^−3^	6.06 × 10^−3^	7.60 × 10^−3^	8.49 × 10^−3^
	Min	1.20 × 10^−4^	1.26 × 10^−4^	2.00 × 10^−4^	2.22 × 10^−4^	3.00 × 10^−4^	3.11 × 10^−4^
**Minimum Principal Strain**	Max	−1.10 × 10^−4^	−8.81 × 10^−5^	−1.00 × 10^−4^	−1.44 × 10^−4^	−2.00 × 10^−4^	−2.04 × 10^−4^
	Min	−1.15 × 10^−3^	−1.92 × 10^−3^	−2.15 × 10^−3^	−3.62 × 10^−3^	−3.24 × 10^−3^	−5.44 × 10^−3^

**Table 8:** Maximum and minimum strain values for geometry B.

		Instant 1		Instant 2		Instant 3	
	Value	DIC	FEM	DIC	FEM	DIC	FEM
**Maximum Principal Strain**	Max	2.76 × 10^−3^	2.73 × 10^−3^	4.28 × 10^−3^	5.38 × 10^−3^	5.10 × 10^−3^	7.58 × 10^−3^
	Min	2.80 × 10^−4^	2.96 × 10^−4^	5.60 × 10^−4^	5.83 × 10^−4^	6.80 × 10^−4^	7.39 × 10^−4^
**Minimum Principal Strain**	Max	−8.00 × 10^−5^	−9.76 × 10^−5^	−1.50 × 10^−4^	−1.90 × 10^−4^	−2.00 × 10^−4^	−2.46 × 10^−4^
	Min	−1.36 × 10^−3^	−1.65 × 10^−3^	−2.58 × 10^−3^	−3.24 × 10^−3^	−3.22 × 10^−3^	−4.77 × 10^−3^
